# Alterations of Gray and White Matter Networks in Patients with Obsessive-Compulsive Disorder: A Multimodal Fusion Analysis of Structural MRI and DTI Using mCCA+jICA

**DOI:** 10.1371/journal.pone.0127118

**Published:** 2015-06-03

**Authors:** Seung-Goo Kim, Wi Hoon Jung, Sung Nyun Kim, Joon Hwan Jang, Jun Soo Kwon

**Affiliations:** 1 Institute of Human Behavioral Medicine, SNU-MRC, Seoul National University Hospital, Seoul, South Korea; 2 Max Planck Institute for Human Cognitive and Brain Sciences, Leipzig, Germany; 3 Department of Psychiatry, College of Medicine, Seoul National University, Seoul, South Korea; 4 Department of Brain and Cognitive Sciences, College of Natural Sciences, Seoul National University, Seoul, South Korea; Bellvitge Biomedical Research Institute-IDIBELL, SPAIN

## Abstract

Many of previous neuroimaging studies on neuronal structures in patients with obsessive-compulsive disorder (OCD) used univariate statistical tests on unimodal imaging measurements. Although the univariate methods revealed important aberrance of local morphometry in OCD patients, the covariance structure of the anatomical alterations remains unclear. Motivated by recent developments of multivariate techniques in the neuroimaging field, we applied a fusion method called “mCCA+jICA” on multimodal structural data of T1-weighted magnetic resonance imaging (MRI) and diffusion tensor imaging (DTI) of 30 unmedicated patients with OCD and 34 healthy controls. Amongst six highly correlated multimodal networks (p < 0.0001), we found significant alterations of the interrelated gray and white matter networks over occipital and parietal cortices, frontal interhemispheric connections and cerebella (False Discovery Rate q ≤ 0.05). In addition, we found white matter networks around basal ganglia that correlated with a subdimension of OC symptoms, namely ‘harm/checking’ (q ≤ 0.05). The present study not only agrees with the previous unimodal findings of OCD, but also quantifies the association of the altered networks across imaging modalities.

## Introduction

Obsessive-compulsive disorder (OCD) is characterized by intrusive, distressing thoughts and ritualistic, repetitive behaviors [1]. A widely accepted neuroanatomical model of OCD suggests the involvement of an abnormal interaction of excitatory and inhibitory cortico-striato-thalamic (CST) pathways [2–4]. Although the original theory was motivated by functional abnormality found in OCD patients [4, 5], several morphological studies reported structural alterations that are relevant to the theory [6–9] A popular computational method called voxel-based morphometry (VBM) [10] has been widely used to assess human brain structures *in-vivo* using magnetic resonance imaging (MRI). Many VBM studies on patients with OCD consistently found aberrant gray matter regional volume in bilateral basal ganglia and dorsal medial frontal cingulate gyri, as summarized by a quantitative meta-analysis [11]. In addition to the gray matter alteration, other studies using diffusion tensor imaging (DTI) found white matter abnormalities in OCD patients, which were localized in corpus callosum [7, 12, 13], cingulum bundles [14] and the white matter in parietal regions [15]. A recent multi-site VBM study including more than 400 patients with OCD also found the aberrant gray and white matter densities in medial and inferior frontal regions [16].

Although the previous studies found structural abnormalities in OCD patients in a significant agreement with the CST hypothesis, the covariance structure of the alterations in gray and white matter remains unclear. In a multimodal anatomical study on pediatric OCD patients [17], three different univariate analyses using T1-weighted MRI and DTI were performed and showed qualitative resemblance among the results. Similarly, a multimodal meta-analysis on brain structures showed larger regional volume of the white matter and smaller fractional anisotropy (FA), which is an index of directionality of a tensor that models water diffusion in the white matter, at the same location of the anterior bundle of corpus callosum in OCD patients than healthy controls [18]. Whereas those two studies showed spatial overlaps of the multimodal alterations [17, 18], another recent multimodal morphological study showed concurrent alterations by constraining one modality by another [19]. In the structural study [19], group differences between OCD patients and healthy controls were found in average cortical thickness of terminal points of the tractography streamlines that were started from white matter voxels with group differences themselves in FA. Despite the qualitative convergence, the association of the structural abnormalities in OCD patients from multiple neuroimaging techniques has not been quantified yet in any other studies to our best knowledge.

In order to quantitatively examine the relationship amongst various alterations that can be measured using different imaging modalities, blind source separation (BSS) methods such as canonical correlation analysis (CCA) and independent component analysis (ICA) have been introduced in multimodal neuroimaging studies [20, 21]. The goal of BSS, under the assumption that the measurements are linear mixtures of independent sources, is to ‘demix’ the measurements (e.g. gray matter density maps) into the latent spatial sources (i.e. structural covariance spanning over certain locations in the brain) and their contributions to the measurements, which are different across individuals [22].

The latent spatial sources from anatomical images reflect the covariance structures in the morphological features, which have been investigated extensively using structural MRI images [23–29]. The covariance may arise from genetic influences, mutual trophic reinforcement, or neuroplasticity based on common experiences [24, 30–32]. Whereas many of the studies used the characteristics of gray matter such as gray matter regional volume [33] or cortical thickness [34], some studies explored the covariance structures of white matter *via* Jacobian determinant of the deformation filed from a non-linear registration, which can be used as a relative measure of the local volume, showing agreements with the manually defined DTI atlas [35, 36]. The Jacobian determinant of white matter was jointly used with concurrent DTI datasets detecting a topological alteration of the developing brain networks [37]. Moreover, ICA-based approaches were also applied to DTI-derived measures such as FA and mean diffusivity (MD) in order to investigate covariance structures in the white matter [38, 39]. The studies demonstrated strong correlations within anatomically meaningful fiber tract bundles [38] and showed the validity of the white matter covariance structures in analyzing the effects of a neurodegenerative disease [39].

The key importance of using multivariate techniques to the multimodal neuroimaging datasets, instead of separate massive-univariate analyses, is the possibility to use cross-information in the multimodal data to explore the complex interplay of brain alterations [40], of which only a specific characteristic (i.e. FA) can be acquired by a certain neuroimaging technique (i.e. DTI). Furthermore, unlike ‘asymmetric’ approaches such as constraining one measurement by the other as in [19], ‘symmetric’ fusion approaches enables researchers to have the multiple datasets jointly contribute to find neurophysiological abnormalities [40], which cannot be achieved by univariate analyses. The fusion techniques have shown their abilities to detect cross-modal abnormalities in patients with schizophrenia [20, 21, 41] and bipolar disorder [42]. Specifically, ICA decomposes naturally separable sources that covary similarly through individuals while it maximizes the statistical independence of sources [43]. On the other hand, CCA finds maximally correlating components between the modalities across subjects [21]. Thus the combination of ICA and CCA has been proposed for natural source separation that is reliable across modalities [42].

In the present study, we applied the combinatory method [42] to find latent covariance patterns of the gray and white matter that contribute to the known structural alterations in the brains of the patients with OCD. It is worthy noting that the multivariate approach does not seek correlation between single voxels but the correlation between spatial sources. Thus using the fusion method, we explored possible relationships between the altered covariance structures in the gray matter and the white matter due to OCD, which may give us an insight to extend the CST theory.

In addition, we further examined whether this fusion method could find multimodal components that correlate with the underlying subscores and subdimensions of the OC symptoms [44]. As the high inhomogeneity of the patients with OCD is well known to the community [44], if we could find a latent component, which is exclusively correlated to a specific subdimension of OC symptoms, it may be useful in differentiating subgroups of the patients and disentangling inhomogeneity of the OC symptoms.

## Materials and Methods

### Human subjects and psychiatric assessment

We recruited 30 patients who fulfilled the criteria for OCD in the fourth edition of the Diagnostic and Statistical Manual of mental disorders (DSM-IV) [1] through the OCD clinic at Seoul National University Hospital (SNUH, Seoul, South Korea). The patients were diagnosed using the Structured Clinical Interview for DSM-IV (SCID). Among the patients with OCD, 22 patients were drug-naïve, and the 8 other patients were unmedicated for at least four weeks at the time of inclusion. Seven patients were assessed to show comorbidity in OCD: three of them to have obsessive-compulsive personality disorders and the four others to have depressive disorder (not otherwise specified).

Along with the patients, we recruited 34 age- and gender-matched healthy controls as well. We used the SCID non-patient version to confirm that none of the controls was with the Axis I psychiatric disorders. The exclusion criteria for both patients and controls included lifetime history of psychosis, bipolar disorder, major depressive disorder, substance abuse or dependence, significant head injury, seizure disorder or mental retardation. All subjects were right-handed. Besides other demographic variables, the intelligence quotient (IQ) was estimated by the Korean-Wechsler Adult Intelligence Scale-Revised (K-WAIS-R). The degrees of depression and anxiety were measured by self-reporting Beck’s Depression Inventory (BDI) [45] and Beck’s Anxiety Inventory (BAI) [46], respectively. The severity of OC symptoms was assessed with the clinician-administered Yale-Brown Obsessive-Compulsive Scale (Y-BOCS) [47]. In addition to the Y-BOCS subscores for obsession and compulsion, we estimated subdimensional scores from Y-BOCS symptom checklist [44, 48], as an alternative measure to quantify subdimensional characteristics of OC symptoms in the absence of multidimensional measures such as such as Dimensional Y-BOCS [49] and Padua Inventory [50]. For the 13 items in Y-BOCS checklist, numerical assessments are given as 0 (absent symptom), 1 (symptom present but not major reason for concern) or 2 (prominent symptom). The subdimensional scores in the present study were simply approximated by the mean scores of certain items as done in [44]: the ‘contamination/washing’ score is the mean of ‘contamination obsession’ and ‘washing/cleaning compulsion’ scores; the ‘harm/checking’ score is the mean of ‘aggressive obsession’ and ‘checking compulsion’ scores; the ‘symmetry/ordering’ score is the mean of ‘symmetry obsession’, ‘checking compulsion’, ‘repeating compulsion’, ‘counting compulsion’ and ‘ordering compulsion’ scores; the ‘sexual/religious obsessions’ score is the mean of ‘sexual obsession’ and ‘religious obsession’ scores; and the ‘hoarding/saving’ score is the mean of ‘hoarding obsession’ and ‘hoarding compulsion’ scores. Y-BOCS checklist scores of four patients were unavailable due to administrative difficulties. Thus correlation analyses on the subdimensional scores were only performed on 26 patients with OCD.

### Ethics statement

The present study was approved by the Institutional Review Board at Seoul National University Hospital (Seoul, South Korea; reference number: C-1405-076-581). All subjects were fully instructed about the scanning and assessment procedures and then submitted written informed consents.

### Imaging acquisition

We obtained T1-weighted 3D MRI using the 1.5 T Magnetom Avanto Syngo scanner (Siemens, Erlangen, Germany) with the following parameters: TR/TE = 1160/4.76 ms, flip angle = 15°, voxel size: 0.45 × 0.45 × 0.90 mm^3^, field of view: 350 × 263 × 350 mm^3^.

We also obtained DTI of the subjects. With 10 repetitions with no diffusion weight, the diffusion weighted images along the 12 noncollinear directions with the b-factor of 1000 s/mm^2^ were acquired with the following parameters: TR/TE = 9200/83 ms, voxel size: 2.0 × 2.0 × 2.0 mm^3^, field of view: 224 × 256 × 150 mm^3^.

T1-weighted MRIs of the current subjects were included in our previous graph-theoretical analysis showing disparity between dorsal and ventral corticocortical networks in the patients with OCD [28], but diffusion-weighted MRIs were not reported in anywhere.

### Multivariate analysis using mCCA+jICA

As we mentioned above, we used a multivariate method that combines multiset-CCA (mCCA) and joint ICA (jICA), called “mCCA+jICA” fusion method [42]. The jICA method has been used for BSS from a multimodal dataset including functional and structural *in-vivo* measurements of human brains [20]. It is noteworthy that, by definition, the context of features is irrelevant to the BSS methods; it is only relevant to the neurobiological interpretations. Therefore the jICA method with/without (m)CCA was applied to functions images with different tasks [51], different structural images [52], and both of functional and structural images [42]. Since the “mCCA+jICA” framework is in a flexible and general form of a BSS method [53], we adopted the framework for the current study.

**Fig 1:**
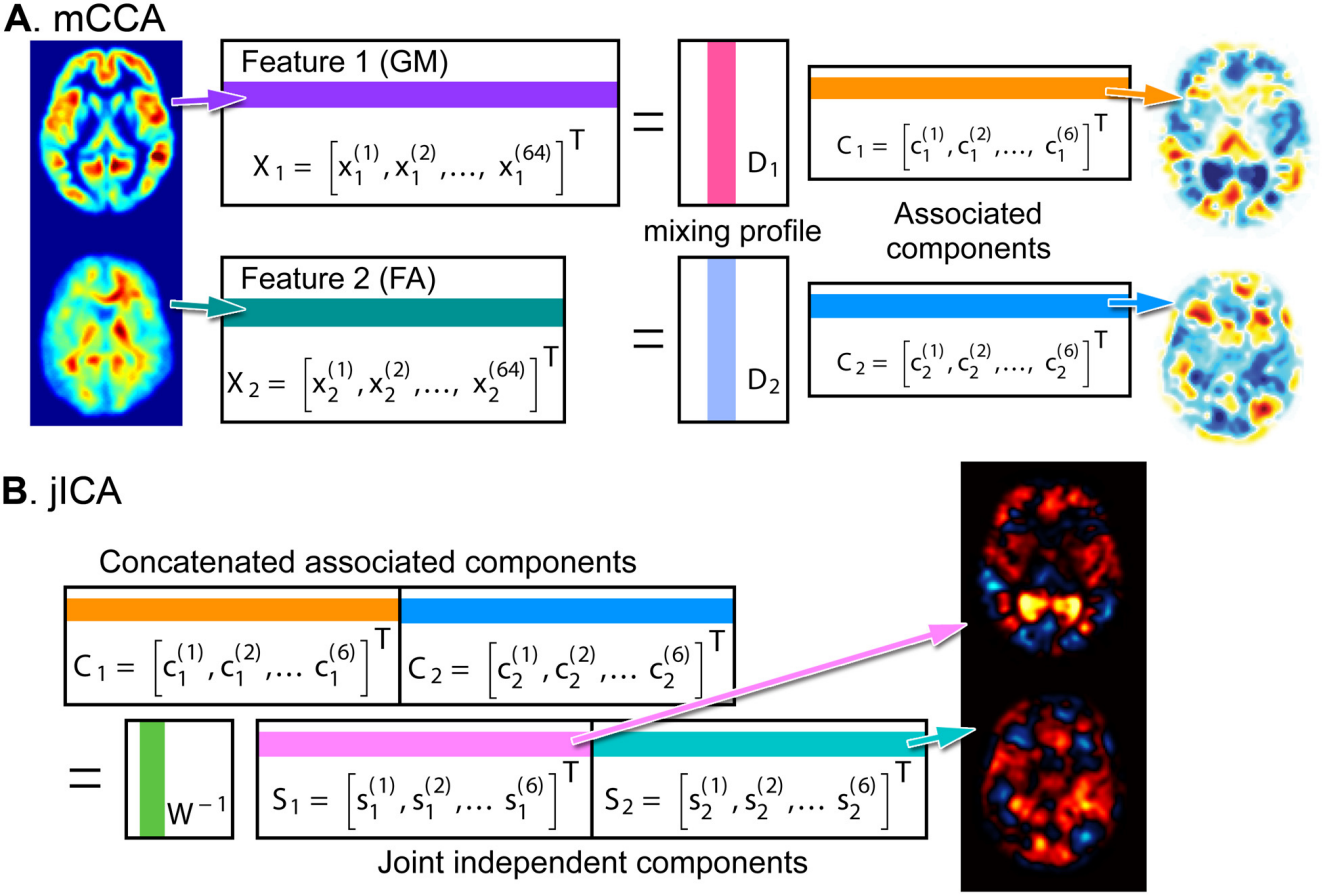
Overview of the fusion method “mCCA+jICA”. The multiset canonical correlation analysis (mCCA, **A**) and joint independent component analysis (jICA, **B**) are depicted. In the mCCA (A), the feature matrix of the k-th imaging **X**_*k*_ with the dimensions of the number of subjects (e.g. 64) by the number of voxels is modeled as a product of mixing profile **D**_*k*_ and associated component **C**_*k*_. Subsequently, in the jICA (B), the concatenated associated component matrix **C**_*k*_ with the dimensions of the number of components (e.g. 6) by the sum of the number of voxels across imaging modalities is modeled by the demixing matrix $W_{k}^{-1}$ and the joint independent components **S**_*k*_. To illustrate the meaning of the row and column vectors in the matrices, the colored vectors in the matrices are back-reconstructed in the brain space and visualized. In the feature matrices X, the i-th row vector of $x_{1}{}^{\left(i\right)}$ and $x_{2}{}^{\left(i\right)}$ are GM and FA images of the i-th subject (A, leftmost). In the associated components matrices C, the j-th row vector of $c_{1}^{\left(j\right)}$ and $c_{2}^{\left(j\right)}$ are source images maximizing inter-modal correlation by mCCA (A, rightmost). In the joint independent component matrix, the l-th row vector $s_{1}^{\left(l\right)}$ and $s_{2}^{\left(l\right)}$ are source images maximizing inter-source independency by jICA (B. rightmost).

The analysis steps of the mCCA+jICA method are illustrated with examples in Fig 1. As the theory is explained in details in the original paper [42], we briefly summarized how we analyzed our data in the following sections.

For the preprocessing of neuroimaging data and the use of probabilistic atlases, FMRIB’s Software Library (FSL; http://www.fmrib.ox.ac.uk/fsl/) was used [54]. For the multivariate analysis of mCCA+jICA [42], Fusion ICA Toolbox (FIT; http://mialab.mrn.org/software/fit/) was employed on the MATLAB environment (Mathworks Inc., Natick, MA, USA). The FIT, which is freely available, provides comprehensive packages for ICA-based fusion analysis. Particularly, it supports interactive graphic user interfaces (GUI) and step-by-step tutorials promoting applications by clinical researchers.

#### Feature selections: preprocessing

The GM image was estimated from the T1-weighted MRI. First the non-brain tissue was discarded using Brain Extraction Tool (BET v2.1) in FSL [55], with the parameters optimized for each subject by means of visual inspection. Then, the tissue types of gray matter, white matter and cerebrospinal fluid were segmented and their probabilities, or concentrations bound between zero and one, within a voxel were estimated using FMRIB's Automated Segmentation Tool (FAST).

The FA computation from the DTI data was carried by FMRIB’s diffusion toolbox (FDT v2.0) in FSL. For each run of DTI scanning, all volumes were corrected for the distortions due to Eddy currents and head motions by affine transformation to the first volume with no diffusion weighting. Then the volumes without diffusion weighting were averaged and non-brain tissue was discarded using BET. Tensor models were fit to describe diffusion in the corrected volumes within the brain mask. Then the directionality of diffusion tensor was computed in terms of FA [56]. The FA would be zero for a perfectly isotropic tensor and one for a perfectly anisotropic tensor.

The resulting GM and FA maps in native space were spatially normalized into Montreal Neurological Institute (MNI) standard space in the resolution of 2-mm isotropic voxel accordingly to the VBM framework [10, 57] as implemented in FSL (FSL-VBM v1.1). We used ‘MNI152-T1’ template for the initial template for the GM images and ‘FMRIB58-FA’ template for the FA images. Study-specific templates for unbiased normalization were created using the linear transformation with FMRIB’s Linear Image Registration Tool (FLIRT) and the following nonlinear transformation with FMRIB’s Non-linear Image Registration Tool (FNIRT). Then the individual images were warped into the study-specific templates for GM and FA, respectively. The registered images were then corrected for inter-subject variability in local scale using the Jacobian determinant of the deformation field, which represents local expansion or contraction [58], as introduced in the ‘optimized VBM’ framework [57]. Because of the correction, the resulting values are no longer bounded between zero and one. The images were smoothed with a Gaussian isotropic kernel with σ of 3 mm, i.e. full-width of half maximum (FWHM) of about 6.9 mm for x-, y-, and z-directions, to reduce the risk of abrupt noise and misalignment during the spatial normalization [59]. We used the preprocessed images for GM and FA as our multimodal features in the following multivariate analysis. The feature maps were scaled in z-scores to make the units of measurements comparable.

#### Dimensionality reduction: multiset-CCA

We modeled multimodal features $X_{k}\in R^{n\times p_{k}}$ as a multiplication of the spatial distribution of sources $S_{k}\in R^{s\times p_{k}}$ and nonsingular mixing matrix A_*k*_ ∊ *R* ^*n×s*^ that represents the contributions of sources to individual images [42] as

$$X_{k}=A_{k}S_{k},$$ (1)

where *k* is an index for modality as *k* = 1 for GM and *k* = 2 for FA, *n* is the number of subjects, *s* is the number of independent sources that is common to both modalities, and *p*_*k*_ is the number of voxels of the modality *k*. The columns of **A**_1_ and **A**_2_ are assumed to be highly correlated only on the corresponding indices [42]. This assumption is much more flexible than a separate method either of multiset-CCA [60] or joint ICA [20], in the sense that the neuroimaging measures are likely to have significant correlations but not necessarily as prefect as previously presumed in joint ICA analysis [20].

As described in Sui and colleagues [42], first the dimensionality of the features was reduced using singular value decomposition (SVD) to the dimensions of 30 for each modality retaining more than 98.9% of non-zero eigenvalues of both modalities. Then we separated the reduced features by the mixing profiles **D**_*k*_∊*R* ^*n×c*^ and the associated components $C_{k}\in R^{c\times p_{k}}$ using mCCA as

$$X_{k}=D_{k}C_{k}.$$ (2)

The mCCA with two features in the present study reduces to CCA [61] similarly to the application in the original paper [42]. The number of associated components was determined as 6 using minimum description length (MDL) criteria [62]. The column vector of **D**_*k*_, called ‘canonical variate’, represents the contribution of the associated component to the features of the individual subjects. The correlation of canonical variates were maximized step-wisely from the first to the last associated component, while the correlation between the canonical covariates with different indices were minimized [61]. The optimized correlations across the modalities were ranged from 0.998 to 0.969 in decreasing order.

#### Spatial decomposition: joint ICA

It is shown that the components in the real brain data found using mCCA are typically contain sources that are not completely decomposed due to the spatial dependency of neuroimaging data across modalities [42]. Therefore, the concatenated (joint) associated components **C = [C_1,_C_2_]** were subsequently separated into spatially independent sources **S = [S_1_,S_2_]** using jICA [20, 42] as

$$C=W^{-1}S,$$ (3)

where *p* = *p*_*1*_ + *p*_*2*_, *s* is the number of independent sources and **W** ^-1^∊*R* ^*c×s*^ is the pseudo-inverse of demixing matrix thus representing the contribution of the independent components (ICs) **S** to individual associated components **C**. The statistical dependency among the joint ICs was minimized using information maximization [63]. Taken together, the method of mCCA+jICA is summarized [42] as

$$X_{k}=D_{k}C_{k}=D_{k}W^{-1}S_{k}=A_{k}S_{k},\;A_{k}=D_{k}W^{-1}.$$ (4)

In other words, the contribution of spatially independent sources to the individual images, which we modeled as **A**_*k*_, is actually given as the linear mixture of the contribution of the independent sources to associated components **D**_*k*_ and the contribution of the associated components to individual images **W** ^-1^.

We used Infomax algorithm to separate independent sources, which performs optimally under the assumption of super-Gaussianess [63]. The super-Gaussianess of the current data, as in many real-world signals [63], was suggested by kurtosis *K* greater than 0 (*K* = 3.33 for GM, *K* = 4.54 for FA). Positive, non-zero kurtosis means that the distribution of the data has a sharper peak and longer tails than the Gaussian distribution. Kolmogorov-Smirnov (K-S) tests also used to confirm that the data is non-Gaussian (*p* < 10^-16^). To increase stability and robustness of the non-linear optimization, the estimates of jICA were performed for 1000 times and averaged.

#### Statistical inferences

Given that the estimated sources are statistically independent and reliable across imaging modalities as possible under the constraint of the given dimensionality, we wish to find some sources that differentially contribute to individual structural images between the OCD patients group and the healthy controls. In order to infer such group differences, two-sample *t-*tests were performed on the IC loadings in the mixing matrix **A_*k*_**. In the context of general linear model (GLM), our model on the IC loading can be written as

$$a_{i,s}=\beta_{0}+\beta_{1}g_{i}+\varepsilon$$ (5)

where *a*_*i*,*s*_ is the mixing coefficient for the *s*-th component of the *i*-th individual, g_*i*_ is a group index being either 0 or 1, *β*_*0*_ and *β*_*1*_ are unknown parameters and *ε* is Gaussian noise with zero mean and unit variance. Since the Gaussian assumption is placed on the error term, we used K-S goodness-of-fit test on the residual of the GLM $a_{i,s}-({\hat{\beta }}_{0}+{\hat{\beta }}_{1}g_{i})$ to justify the normality of the mixing coefficients [64].

Moreover, as we introduce a multiple comparisons problem by collectively performing multiple *t*-tests for 12 independent sources, False Discovery Rate (FDR) was applied to control the family-wise type-I error level, i.e. *q* = 0.05 [65].

In addition to the group-wise analysis, we examined correlations between the clinical measures and the IC loadings within the OCD patients. Spearman’s rank correlation was used in order to minimize sensitivity to outliers. In the correlation analysis, the mixing coefficients of patients describe the contributions of a group-specific component, which is distinct from the common sources **S**_*k*_ we mentioned above [42]. To obtain a group-specific source, the mixing matrix **A**_*k*_ and features **X**_*k*_ in (Eq 1) were separated into the healthy controls (HC) and the OCD patients as

$$X_{k}=\left[\begin{array}{c} X_{\text{HC},k}\\ X_{\text{OCD},k} \end{array}\right],\;A_{k}=\left[\begin{array}{c} A_{\text{HC},k}\\ A_{\text{OCD},k} \end{array}\right].$$ (6)

Then the group-specific sources are given as

$$S_{\text{HC},k}=A_{\text{HC},k}^{-1}X_{\text{HC,k}},\;S_{\text{OCD},k}=A_{\text{OCD},k}^{-1}X_{\text{OCD,k}},\;k=1,2.$$ (7)

Including irrelevant variables as covariate terms in the GLM might introduce reduce precession in estimating unknown coefficients [66]. Thus we did not covary any other factors in GLMs and correlation analysis such as age, sex, the age of onset of illness, the duration of illness, the depression level (BDI), and the anxiety level (BAI) as none of them was significantly correlated with the IC loadings (*q* > 0.05). Although we found significant difference in the BDI and BAI between the patients and the healthy controls (see the Results section below), those variables were not correlated with the loading coefficients of the ICs, thus not included as covariates.

To identify the anatomical dispositions of the components, the probabilistic atlases of FSL were employed. Specifically, Harvard-Oxford cortical [67] and subcortical structural [68] atlases were used for gray matter, and Johns Hopkins University white-matter tractography atlas [69] was used for white matter. For each cluster, brain regions with maximal probabilities were determined based on the mean probability of a certain label in the atlases. When the white matter clusters cannot be identified using white-matter tractography atlas, the proximate gray matter structures were used to describe anatomical locations.

## Results

### Demographic and clinical variables

The demographic and the clinical variables of the current subjects are given with test statistics and *p*-values for the equality of group means in Table 1. There were no significant differences found in age (*p* = 0.396), gender ratio (*p* = 0.935) and education year (*p* = 0.662) and IQ (*p* = 0.658) suggesting the controls were well matched in the demographic variables. The patients with OCD showed significantly different levels of depression and anxiety in relation to the healthy controls: BDI (*p* < 10^-7^) and BAI (*p* < 10^-6^) scores were significantly higher in the OCD patients than in the healthy controls. The mean of the total Y-BOCS scores of the OCD patients was 21.17 with the standard deviation of 6.24.

**Table 1:** Demographic and clinical variables of patients with OCD.

Variable	OCD patients (n = 30)	Controls (n = 34)	*t-*/*z*-stat.	*p*-value
Age (year)	25.00 ± 6.57	23.88 ± 3.63	0.86	0.395
Gender (m/w)	20 / 10	23 / 11	-0.05	0.961
Education (year)	13.30 ± 3.31	14.04 ± 1.31	0.44	0.662
IQ	112.17 ± 11.24	113.35 ± 10.09	-0.45	0.658
BDI	17.70 ± 10.90	4.00 ± 6.08	6.31	< 10^-7^
BAI	18.83 ± 14.15	4.35 ± 5.46	5.52	< 10^-6^
Y-BOCS				
Obsession	12.07 ± 3.58	-	-	-
Compulsion	9.10 ± 4.84	-	-	-
Total	21.17 ± 6.24	-	-	-
Age of onset (year)	16.20 ± 7.35	-	-	-
Duration of illness (year)	7.67 ± 6.73	-	-	-

Mean and standard deviation are given as ‘mean ± std.’, except for gender ‘men/women’. Test statistics for group differences between the patients and controls are given with *p*-values. Abbreviations: BDI, Beck’s Depression Inventory; BAI, Beck’s Anxiety Inventory; Y-BOCS, Yale-Brown Obsessive-Compulsive Scale.

The numbers of patients with present (score = 1) and prominent (score = 2) symptoms of Y-BOCS checklist [47] and the numbers of patients with present (0 < score ≤ 1) and high (1 < score ≤ 2) scores of five subdimensional scores [48] are given in Table 2 (n = 26). As there were small (≤ 4) patients with subdimensional scores greater than zero for ‘sexual/religious obsessions’ and ‘hoarding/saving’, we only used the three subdimensional scores of ‘contamination/washing’, ‘harm/checking’ and ‘symmetry/ordering’ in the following correlation analysis. The mean and standard deviation of the analyzed scores were: ‘contamination/washing’, 1.06 ± 0.92; ‘harm/checking’, 0.56 ± 0.54; ‘symmetry/ordering’, 0.32 ± 0.29. The pair-wise rank correlations between subdimensional scores were not significant in any pairs (min *p* = 0.181) as the orthogonality of subdimensions was suggested in a previous factor analysis on Y-BOCS checklist [48].

**Table 2:** The numbers of patients with present and prominent symptoms categorized by the Y-BOCS checklist and the estimated subdimensional scores (n = 26).

Y-BOCS checklist items	Present (= 1)		Prominent (= 2)	
Obsessions				
Aggressive	2	(8%)	4	(15%)
Contamination	1	(4%)	14	(54%)
Sexual	1	(4%)	1	(4%)
Hoarding	1	(4%)	1	(4%)
Religious	2	(8%)	1	(4%)
Symmetry	4	(15%)	0	(0%)
Somatic	1	(4%)	3	(12%)
Compulsions				
Washing	2	(8%)	12	(46%)
Checking	3	(12%)	8	(31%)
Repeating	8	(31%)	10	(38%)
Counting	1	(4%)	1	(4%)
Ordering	2	(8%)	0	(0%)
Hoarding	1	(4%)	1	(4%)
Estimated subdimensional scores	Present (0 < score ≤ 1)		Prominent (1 < score ≤2)	
Contamination/washing	4	(15%)	12	(46%)
Harm/checking	15	(58%)	1	(4%)
Symmetry/ordering	17	(65%)	0	(0%)
Sexual/religious obsessions	4	(15%)	0	(0%)
Hoarding/saving	1	(4%)	1	(4%)

### Joint independent components with group differences

**Fig 2:**
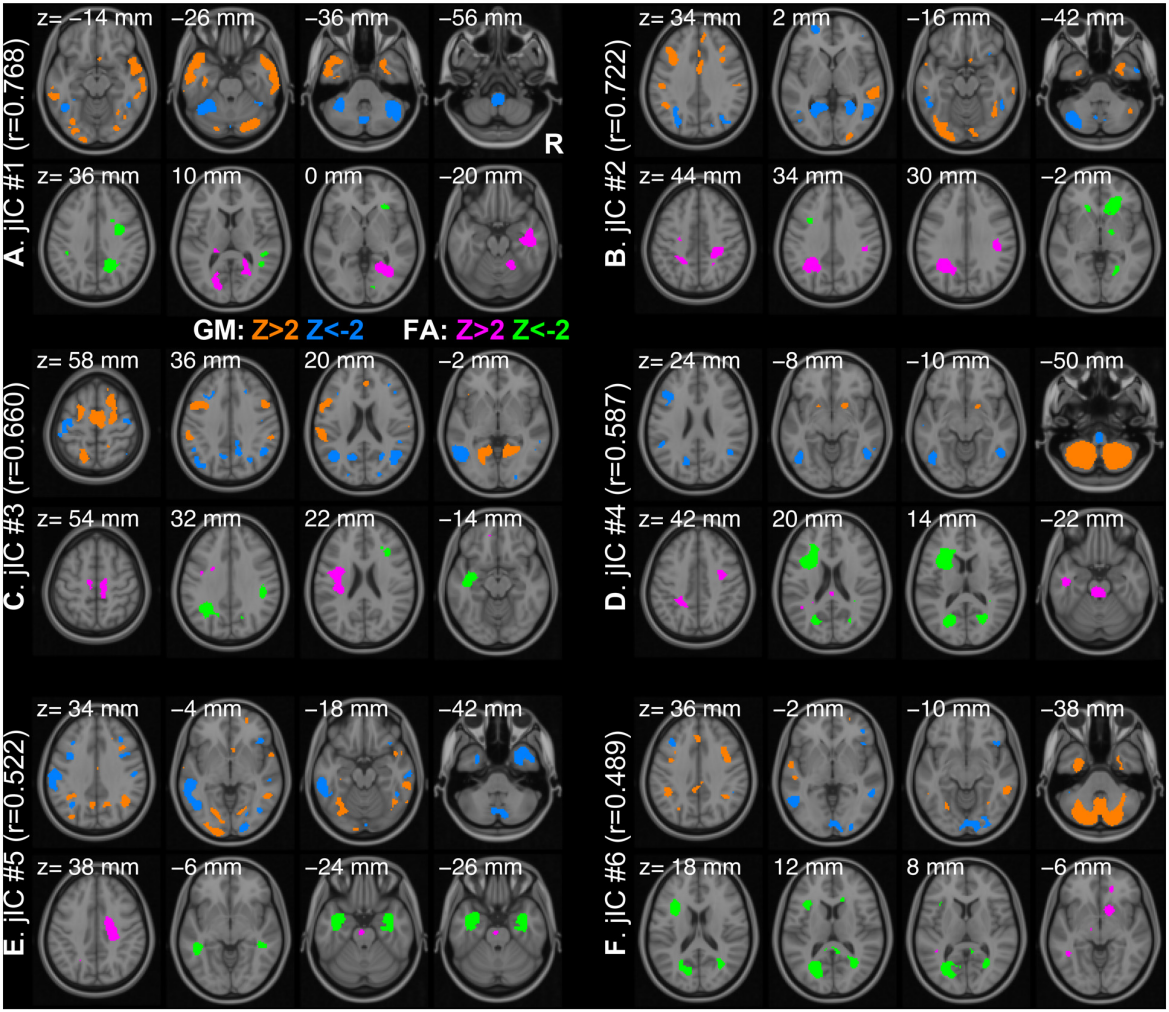
Six joint independent components. Back-reconstructed maps of the components are shown with a threshold of |z| > 2 (A-E) for GM (orange, *z* > 2; blue, *z* < -2) and FA (magenta, *z* > 2; green, *z* < -2). The threshold is not chosen for a statistical significance but only for visualization of the components. The Pearson’s correlation between the loading coefficients of GM and FA are also noted in parentheses. The axial slices are oriented in a neurological convention (the right hemisphere is on the right side of the image) and chosen for the largest four clusters. The MNI z-coordinate is noted on the top of each slice.

Using the mCCA+jICA framework, we found six joint independent components **S**_*k*_ for each modality as we determined by MDL criteria. The components were highly correlated across modalities in terms of their contribution to the individual images (i.e. loading coefficients). The highest Pearson’s correlation of the first joint components was 0.768 and the lowest one of the sixth joint components was 0.489 (*p* < 10^-4^). All joint ICs were back-reconstructed in the brain space, which are visualized with the threshold of |z| > 2 in Fig 2. Notably, the threshold of |z| = 2 was not chosen for a statistical significance but only arbitrarily selected for the visualization of ICs. One may find that some components are remarkably localized such as GM #4 (inferior parts of the bilateral cerebella) and FA #5 (the bilateral inferior longitudinal fasciculi). Many of multimodal networks, however, span over remote brain regions showing long-range associations of the local morphometric features.

To validate the Gaussianess of the mixing coefficients, K-S tests for the 12 IC loadings were carried out on the residuals of the GLM (Eq 5). As none of the K-S tests rejected the hypothesis on the normality (min uncorrected *p* = 0.099), we proceeded to use the *t-*test to infer group differences.

**Fig 3:**
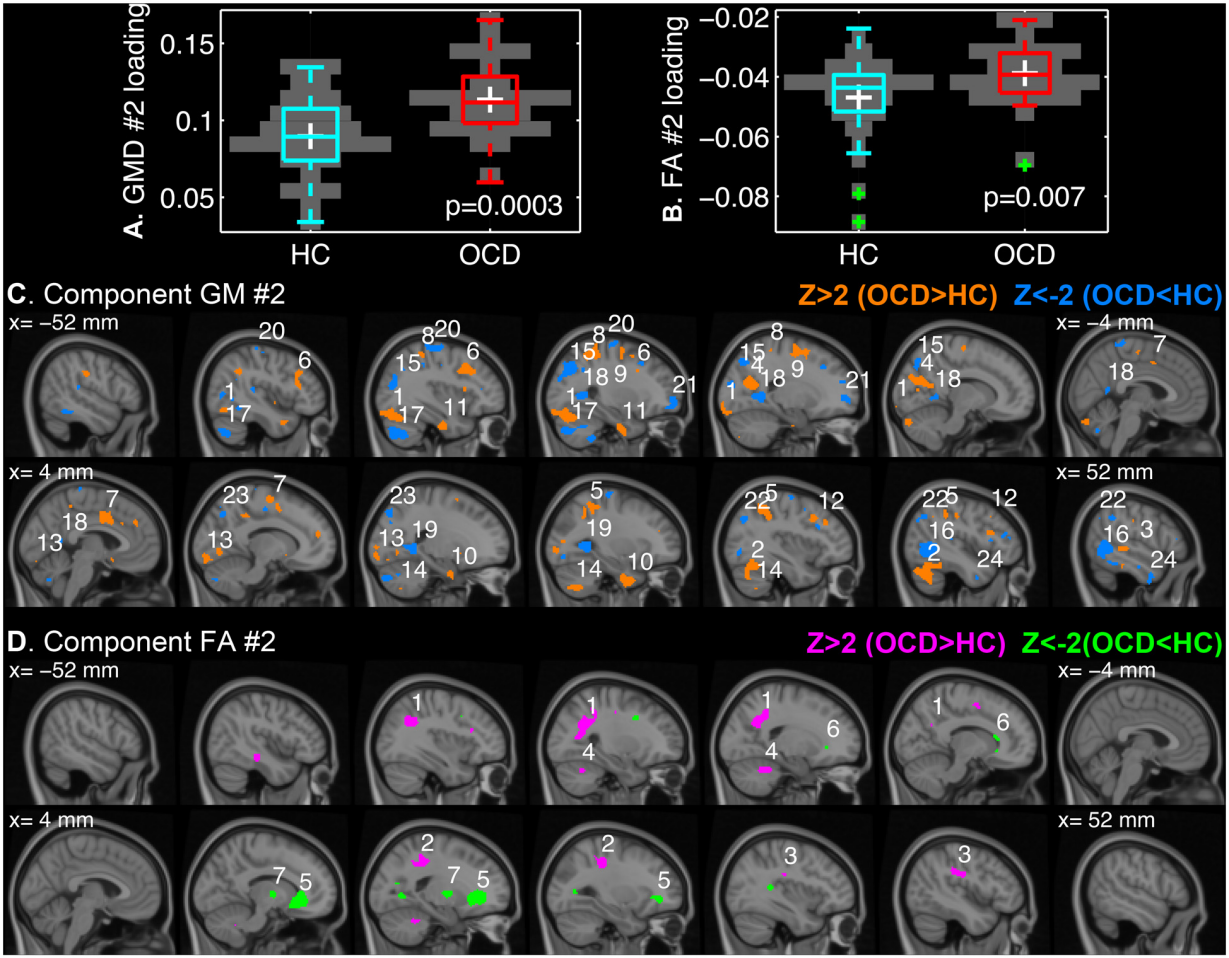
The second joint independent components (IC) that differed between the patients with OCD and the healthy controls. The loading coefficients of GM #2 (A) and FA #2 (B) are given in boxplots overlaid on discrete violin-plots with the *p*-values of *t-*tests for group differences. The *z*-transformed IC maps are visualized over the sagittal slices of MNI152 template from the x-coordinate of -52 mm (left hemisphere) to 52 mm (right hemisphere) for every 8 mm with a threshold of |z| > 2 for GM #2 (C) and FA #2 (D). The number on each cluster denotes the cluster index that can be found in Table 3.

We found that the second joint ICs (GM #2 and FA #2) differentially contributed to the individual images between the OCD patients and the controls (*q* ≤ 0.05; GM #2, *p* = 0.0003; FA #2, *p* = 0.007). The boxplots of the IC loadings and the spatial distributions of the sources are shown in Fig 3. For the suprathreshold clusters, most probable anatomical annotations, peak *z*-statistics and MNI-coordinates can be found in Table 3.

**Table 3:** Suprathreshold clusters of the second joint independent components that differed between the patients with OCD and the healthy controls.

Cluster index	Volume (cm^3^)	Peak z-stat.	MNI-coordinate (mm)	Anatomical region
**A.** GM #2 (Positive z-value, OCD > HC; Negative z-value, OCD < HC)				
1	6.54	3.96	(-32, -78, -16)	Left cerebellum and left occipital fusiform gyrus and left lateral occipital cortex (inferior)
2	5.45	3.51	(40, -60, -16)	Right cerebellum and right temporal occipital fusiform cortex
3	3.77	4.16	(58, -32, 0)	Right middle temporal gyrus (posterior) and right superior temporal gyrus (posterior)
4	3.71	5.89	(-20, -62, 18)	Left precuneous cortex and left cuneal cortex and left supracalcarine cortex
5	3.66	4.18	(34, -42, 42)	Right superior parietal lobule
6	3.28	5.43	(-34, 14, 36)	Left middle frontal gyrus
7	3.26	3.38	(4, 4, 40)	Right cingulate gyrus (anterior) and left cingulate gyrus (anterior) and right supplementary motor cortex
8	2.80	4.78	(-30, -38, 54)	Left superior parietal lobule and left postcentral gyrus
9	2.74	3.14	(-24, -14, 58)	Left superior gyrus and left precentral gyrus
10	2.30	2.94	(28, -4, -38)	Right parahippocampal gyrus (anterior) and right temporal fusiform cortex (anterior) and right temporal pole
11	1.66	3.26	(-32, -10, -34)	Left temporal fusiform cortex (anterior) and left temporal fusiform cortex (posterior) and left parahippocampal gyrus (anterior)
12	1.33	5.21	(34, 14, 38)	Right middle frontal gyrus
13	1.32	2.62	(16, -92, -10)	Right occipital pole and right occipital fusiform gyrus and right lingual gyrus
14	1.20	2.91	(28, -66, -48)	Right cerebellum
15	6.86	-4.07	(-32, -60, 34)	Left lateral occipital cortex (superior)
16	6.06	-5.16	(48, -58, 2)	Right lateral occipital cortex (inferior) and middle/inferior temporal gyri (temporooccipital)
17	4.73	-5.42	(-34, -70, -42)	Left cerebellum
18	3.30	-4.50	(-24, -52, 4)	Left lingual gyrus and precuneous cortex
19	2.43	-3.93	(26, -50, 4)	Right lingual gyrus
20	2.18	-3.30	(-30, -16, 68)	Left pre/postcentral gyri
21	1.76	-3.55	(-22, 54, 0)	Left frontal pole
22	1.71	-2.87	(32, -70, 28)	Right lateral occipital cortex (superior)
23	1.28	-3.05	(20, -80, 44)	Right lateral occipital cortex (superior) and precuneous cortex
24	1.07	-2.68	(48, 0, -36)	Right inferior and middle temporal gyri (anterior)
**B.** FA #2 (Positive z-value, OCD > HC; Negative z-value, OCD < HC)				
1	7.20	4.72	(-32, -54, 34)	Left superior longitudinal fasciculus
2	2.77	2.92	(30, -32, 44)	White matter near right postcentral gyrus
3	1.25	2.77	(44, -28, 30)	Right superior longitudinal fasciculus
4	1.22	2.69	(-20, -46, -30)	White matter in left cerebellum
5	6.78	-3.73	(18, 32, -2)	Forceps minor
6	1.18	-2.27	(-16, 24, -8)	White matter near left caudate
7	1.17	-3.00	(16, -6, 2)	White matter near right pallidum and thalamus

Only clusters with peak |z| > 2 and larger volume than 1 cm^3^ are tabulated for simplicity.

It is noteworthy that the three subjects with low FA #2 loadings (Fig 3B, green crosses) did not drive the group difference. In fact, the *p*-value of the two-sample *t-*test decreases if the subjects are discarded (*p* = 0.003). However, we did not regard the subjects as outliers to be excluded from the analysis because none showed critically high inter-subject variability in terms of the all IC loadings. Specifically, the mean of each column of the covariance matrix of the IC loading across the subjects was higher than the overall mean subtracted by two standard deviations for all individuals. A similar approach was introduced to assess the homogeneity of GM maps in VBM8 toolbox (http://dbm.neuro.uni-jena.de/vbm/check-sample-homogeneity/).

The mixing coefficients of the joint ICs were higher in the OCD patients than the controls (Fig 3A and 3B). It can be interpreted as, in the OCD patients with the higher coefficients, the corresponding component had greater contribution to the original feature than the controls in a region with positive *z*-value of the IC map. On the other hand, the contribution in the patients was smaller in a location with the negative z-value of the IC maps [20]. The GM #2 component was widely distributed over the cerebella, bilateral middle temporal gyri, inferior occipital lobes, superior parietal lobules and middle frontal gyri, and cingulate gyri (Fig 3C). On the other hand, the FA #2 component was localized on the superior longitudinal fasciculi, forceps minor, and the white matter in subcortical structures (Fig 3D).

### Correlation analysis with IC loadings and clinical measures

Subsequently, we analyzed the correlations between the IC loadings and the clinical measures within the OCD patients. We did not find any significant rank correlations of the mixing coefficients with the Y-BOCS total and subscores (*q* > 0.05). Uncorrected *p*-values were smaller than 0.05 for negative rank correlations of the GM #1 coefficients with Y-BOCS total scores and compulsion subscores (min *p* = 0.012). But none survived after the FDR was applied.

**Fig 4:**
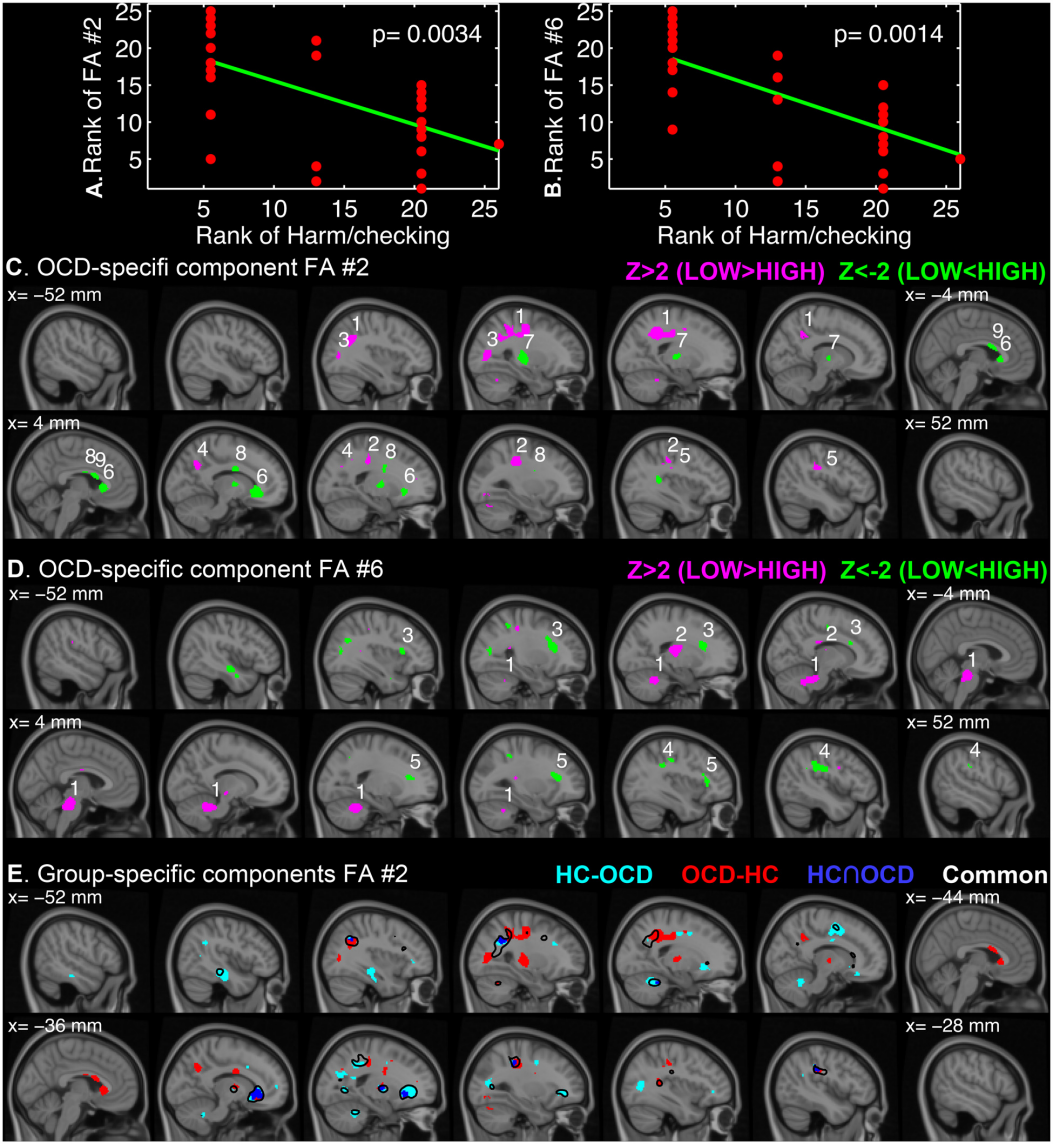
OCD-specific independent components (IC) that correlated with a subdimensional score of OC symptom. The ranks of IC loadings of OCD-FA #2 (A) and OCD-FA #6 (B) are plotted over the ranks of ‘harm/checking’ subdimensional scores. The *z*-transformed IC maps are visualized over the sagittal slices of MNI152 template from the x-coordinate of -52 mm (left hemisphere) to 52 mm (right hemisphere) for every 8 mm with a threshold of |z| > 2 for OCD-FA #2 (C) and OCD-FA #6 (D). The number on each cluster denotes the cluster index that can be found in Table 4. To illustrate the differences of the common and group-specific FA #2 components (E), the set differences (HC—OCD, cyan; OCD—HC, red) and the intersection (OCD ∩ HC, blue) of the group-specific maps with the common map (black contour) are shown.

On the other hand, we found significant rank correlations between the IC loadings and a subdimensional score of the OC symptoms (*n* = 26, *q* ≤ 0.05). As given in Fig 4, the second and sixth OCD-specific white matter ICs were found to negatively correlate with subdimensional scores of ‘harm/checking’, respectively (OCD-FA #2, Spearman’s *r* = -0.554, *p* = 0.003; OCD-FA #6, Spearman’s *r* = -0.594, *p* = 0.001). For the suprathreshold clusters, the most probable anatomical annotations, peak *z*-statistics and MNI-coordinates are tabulated in Table 4.

**Table 4:** Suprathreshold clusters of the OCD-specific independent components that correlated with a subdimensional score of OC symptom.

Cluster index	Volume (cm^3^)	Peak z-value	MNI-coordinate (mm)	Anatomical region
**A.** OCD-specific FA #2 (Negative correlation with ‘harm/checking’ subdimension)				
1	9.62	4.76	(-26, -46, 42)	Left corticospinal tract
2	2.72	3.46	(30, -32, 42)	Right superior longitudinal fasciculus
3	1.78	3.22	(-30, -72, 10)	Left inferior longitudinal fasciculus, forceps major and left inferior fronto-occipital fasciculus
4	1.14	3.52	(14, -62, 34)	White matter near right precuneous cortex
5	1.05	3.20	(46, -28, 32)	Right superior longitudinal fasciculus
6	4.65	-3.60	(8, 24, 0)	Forceps minor
7	2.30	-2.62	(-26, -20, 6)	White matter near left putamen and thalamus and left corticospinal tract
8	1.78	-2.63	(12, -4, 28)	White matter near right caudate
9	1.08	-2.77	(2, 10, 18)	White matter near left cingulate gyrus (anterior)
**B.** OCD-specific FA #6 (Negative correlation with ‘harm/checking’ subdimension)				
1	11.27	3.69	(20, -48, -30)	White matter in brain-stem
2	2.43	2.94	(-22, -20, 14)	White matter near left thalamus and left corticospinal tract
3	3.53	-2.81	(-28, 20, 18)	Left anterior thalamic radiation
4	2.57	-3.32	(46, -28, 32)	Right superior longitudinal fasciculus
5	2.54	-2.70	(30, 22, 18)	Right anterior thalamic radiation

Only clusters with peak |z| > 2 and larger volume than 1 cm^3^ are tabulated for simplicity.

Since the correlations were negative, a positive *z*-value (Fig 4C and 4D; magenta) implies lower contributions in the patients with low ‘harm/checking’ scores than ones with high scores whereas a negative *z*-value (Fig 4C and 4D; green) indicates higher contributions in the patients with the high scores of the subdimension of OC symptom. The OCD-specific FA #2 component was mainly localized on the left corticospinal tract as well as forceps minor (Fig 4C). The OCD-FA #6 component spanned over the brainstem, bilateral anterior thalamic radiation, and the white matter near the left thalamus (Fig 4D).

It should be noted that the group-specific IC maps have different spatial dispositions from the common IC map. In order to illustrate the degree of divergence between the common FA #2 and the group-specific FA #2 components, the thresholded (|z| > 2) set differences from each other (HC-minus-OCD in cyan, OCD-minus-HC in red), and the intersection (blue) are visualized in Fig 4E. The common FA #2 components is also overlaid in Fig 4E as black contours with |z| = 2. The OCD-minus-HC set, i.e. exclusively OCD-specific regions, included a distinctive extension to the left superior longitudinal fasciculus toward the anterior and posterior directions and the focal clusters on the anterior parts of the corpus callosum. Therefore, the finding of the negative correlations of the OCD-specific FA #2 loadings with the ‘harm/checking’ subdimension should be differentiated from the finding of the common FA #2 with the group difference.

## Discussion

### Advantages of the multivariate fusion method over separate univariate analyses of multimodal data

**Fig 5:**
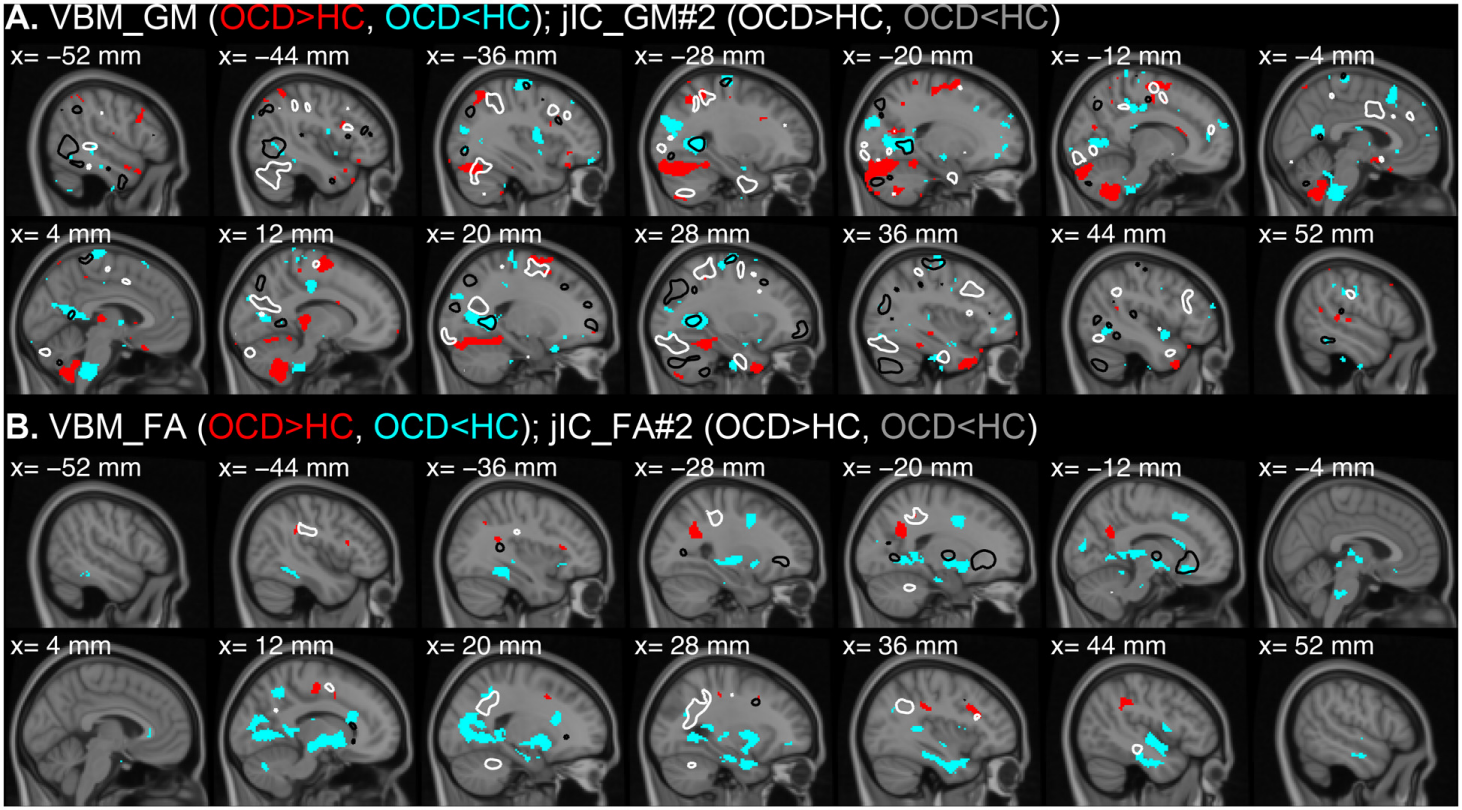
Comparison between VBM results and mCCA+jICA results. VBM results comparing GM (A) and FA (B) between the OCD patients and controls are visualized over sagittal slices from the x-coordinate of -52 mm (left hemisphere) to 52 mm (right hemisphere) with a threshold of |*t*| > 1.99, which corresponds uncorrected *p*-value < 0.05. For both GM and FA, the regions with higher values in OCD than HC are shown in red and the opposites are shown in cyan. The GM #2 (A) and FA #2 (B) components showing significant group differences are superimposed in white (Z = 2, OCD > HC) and black (Z = -2, OCD < HC) contours.

In order to illustrate the utility of the mCCA+jICA method, the joint IC maps (|z| > 2) are compared with the separate VBM analyses on the gray and white matter in Fig 5. Despite major agreements such as the bilateral lingual gyri (Fig 5A, x = -28, -20, 20, 28 mm) and the left region of forceps minor (Fig 5B, x = -20, -12 mm), the discrepancy between two analyses was noticeable. One possible reason is the spatial configuration of the sources. In case a source with a group difference overlaps another common source to a large extent, the difference between the univariate and multivariate analyses can be exaggerated (a detailed discussion with a simulation is provided in S1 File). But more importantly, the difference between VBM and mCCA+jICA results comes from the complexity of the covariance structures. That is, because multiple components affect the intensity of a certain voxel, a difference in the contribution of a certain source is not necessarily apparent from the mixture of all sources.

For an interesting example, a gray matter abnormality (OCD<HC) in the brainstem found by VBM (Fig 5A, x = -4, 4 mm) did not overlap the IC of GM #2, which showed aberrant mixing coefficients in the OCD patients. On the other hand, a similar gray matter abnormality (OCD<HC) in the bilateral lingual gyri found by VBM (Fig 5A, x = -28, 28 mm) exactly converges to the IC of GM #2 (Fig 3C, cluster #17/18). That is, by means of the mCCA+jICA analysis, it was shown that the brainstem abnormality was not related to the lingual gyral abnormality and other major regions of the joint ICs (GM #2 and FA #2). As shown here, it is demonstrated that the multivariate analysis can provide additional information about the interrelationship of the abnormalities.

### Altered structural networks in OCD patients

Using the mCCA+jICA framework, we found one altered pair of cross-modal networks of neuronal structures in the OCD patients in relation to the healthy controls. To our best knowledge, the current paper is the first study that reports the covariance structures of anatomical alterations in the unmedicated patients with OCD using a multivariate fusion method on multimodal neuroimaging data. The second joint components (GM #2 and FA #2) showed significant cross-modal alteration with high congruence in terms of spatial dispositions. As expected, the alterations in the frontal regions were found in the GM #2 (Fig 3C, cluster #6/7/9/12/21) and the FA #2 (Fig 3D, cluster #5). The altered gray matter network (GM #2) extended to the dorsolateral prefrontal cortex (cluster #6/12) [70], frontal pole (cluster #21) [71], and the cingulate cortex (cluster #7) [72], which were also reported in previous univariate studies. In addition, we also found the anomaly in the forceps minor, which has been consistently found to be deviant from the healthy controls in adult and adolescent patients with OCD [12, 15, 17–19, 73–77]. In particular, our group previously demonstrated smaller FA values in the fronto-callosal projections in unmedicated OCD patients based on a fiber bundle tractography [13]. In the present study, we also found irregularities in the anterior part of corpus callosum as well as the subcortical projections in congruence with the white matter abnormalities in the previous findings. Moreover, the FA #2 network included the white matter near the right pallidum and thalamus, which might be directly related to the striatal connectivity in the CST theory. Our present findings not only agree with the previous literature, but also quantitatively connect the abnormalities in the gray and white matter.

It should be also noted that the second joint components involve other brain regions than the “classical” frontal cortex and subcortical structures that have been emphasized by the CST hypothesis [4]. The GM #2 component showed alteration in the bilateral occipital cortices including lingual gyri (Fig 3C, GM #2, cluster #15/18) and the FA #2 component showed white matter abnormality in the bilateral superior longitudinal fasciculi nearby the gray matter alteration (Fig 3D, FA #2, cluster #1). The abnormality of the occipital cortex was previously found in terms of a local surface area in the drug-naïve OCD patients [78]. The disorganization of superior longitudinal fasciculi implicated by smaller FA values was also reported in previous DTI studies [77, 79]. The possible involvement of occipital lobes was mentioned with the commonly known clinical observation of that OCD patients are disturbed by vivid, intrusive visual imagery with unpleasant contents and the common deficits of OCD patients in decision-making and visuospatial tasks [79].

Furthermore, the GM #2 extended to the parietal cortices [7, 72, 80], the temporal cortices [44, 81] and the cerebella [7, 8, 81] in favor of the reconceptualization of the underlying mechanism of OCD with the growing bodies of neuroimaging evidences [82]. Especially, we found the multimodal components spanning the large area of cerebella, in which the multi-site VBM study reported higher regional volume of the gray matter [16]. Besides the well-known functionality of the cerebellum in a fine adjustment of motor actions, the involvement in cognitive and emotional processes has been proposed based on anatomical and functional neuroimaging studies with clinical and healthy populations [83, 84]. More recently, using a surface-based morphometry, the local volume of the cerebellum was found to positively correlate with the comorbidity of OCD in the patients with Tourette syndrome [85]. Taken together, the cerebellar aspect of the joint components may have an implication of altered engagement in the OC symptoms.

### Differential correlations with the subscores and subdimensions of OC symptoms

Within the OCD patients, we did not find any significant rank correlations of the mixing coefficients with the Y-BOCS total score and the subscores of obsession and compulsion, respectively (*q* > 0.05). However, we found significant rank correlation with the ‘harm/checking’ subdimensional score (OCD-FA #2 and OCD-FA #6; *q* ≤ 0.05).

As noted earlier, the group-specific components should be distinguished from the common components since they could be spatially different and work as different bases. The common FA #2 behaved as a source that distinguish the OCD patients from the healthy controls while the OCD-FA #2 served as a source that can differentiate the patients with considerable severity in ‘harm/checking’ subdimension from the other patients with different subtypes. This can be explained by the differences in spatial configuration of the common and the group-specific ICs (Fig 4E).

The contributions to the white matter near the left putamen, the left thalamus and the right caudate (Fig 4C, OCD-FA #2, cluster #7/8) and the contributions to the bilateral anterior thalamic radiations (Fig 4D, OCD-FA #6, cluster #3/5) were heightened with the increasing ‘harm/checking’ subdimension. The subcortical involvement has been considered as crucial in the pathogenesis of OCD [4]. In a mice model, cortico-striatal stimulation repeated over several days evoked OCD-like behaviors that prolonged more than two weeks after the termination of stimulation [86]. Even more directly, the functional role of the associate-limbic area and the subthalamic nucleus in checking behavior of OCD patients was recently demonstrated using microelectrode recording during a surgery for deep brain stimulation [87]. We believe our findings of the correlations of the OCD-specific ICs including the striatal projections (OCD-FA #2) and the thalamic radiation (OCD-FA #6) with the ‘harm/checking’ subdimension reflect the altered organization of the subcortical connectivities in particular association with the subdimension of OC symptoms.

Finally, we also found lower contribution to the brainstem (Fig 4D, OCD-FA #6, cluster #1) and the left corticospinal tract (Fig 4C, OCD-FA #2, cluster #1; Fig 4D, OCD-FA #6, cluster #2) with the increasing ‘harm/checking’ subdimension. Although there are few neuroimaging studies reporting the alterations in the brainstem and its projection [17, 88], Gilbert and colleagues found the regional volume of the gray matter in the left midbrain negatively correlating with the severity of checking behavior [88], which can be related to the current finding of the negative correlation between the OCD-specific white matter component and the ‘harm/checking’ subdimension.

### Limitations

Although our study provides novel findings on the structural networks in the OCD patients using an advanced multimodal method, it also bears some limitations. First, in contrast to the previous structural studies on the Schizophrenia patients using an ICA-fashion multivariate method [43, 52, 89], which employed hundreds of subjects, the sample size of the current study is relatively limited. Since the higher degrees of freedom can be beneficial in decomposing biologically interpretable bases, not only the robustness but also the validity of the current findings can be even improved with a greater number of samples. A recent multi-site VBM study can be an example to cope with the scarcity of the clinical samples [16], although additional caution and elaborated techniques must be accompanied.

Second, a detailed assessment of OC symptoms, such as Dimensional Y-BOCS [49], Obsessive-Compulsive Inventory—Revised [90] and Padua Inventory [50], was unavailable for the current neuroimaging dataset. Since the importance of differentiating multiple dimensions of the OC symptoms has increasingly drawn attention in order to deal with the heterogeneity of the clinical population, it is critical to quantify the symptoms into multivariate measures rather than summing the all details into a representative scalar measure [44, 82]. Therefore it is strongly desirable to include an exhaustive battery of symptom assessments in future multimodal neuroimaging studies.

### Conclusion

In summary, we investigated gray and white matter structural networks in the OCD patients using the fusion method called “mCCA+jICA”. From GM and FA features, the six joint independent components were found, which highly correlated across imaging modalities. A pair of gray and white matter networks spanning over the occipital and parietal cortices, the corpus callosum and the cerebella was found to be altered in the patients with OCD in terms of the contribution to the morphological measurements. Moreover, a particular subdimensional score of OC symptoms was correlated with the loading coefficients of two OCD-specific white matter components including subcortical projections. In future works, the present fusion method can be more useful with a larger sample size and comprehensive subdimensional scores to disentangle the complex pathology of OCD [91].

## Supporting Information

S1 File Simulations for plausible underlying neurobiological structures. (PDF)
