# Supplementary material for: Alterations of Gray and White Matter Networks in Patients with Obsessive-Compulsive Disorder: A Multimodal Fusion Analysis of Structural MRI and DTI Using mCCA+jICA
Source: PLoS One. 2015 Jun 3;10(6):e0127118. doi: 10.1371/journal.pone.0127118 (PMC4454537; doi:10.1371/journal.pone.0127118)
Supplement: S1 File — (PDF) [file pone.0127118.s001.pdf]

Supporting Information

Title: Alterations of gray and white matter networks in patients with obsessive-compulsive disorder: A multimodal fusion analysis of structural MRI and DTI using mCCA+jICA

Authors: Seung-Goo Kim [1,2], Wi Hoon Jung [3], Sung Nyun Kim [4], Joon Hwan Jang [4] and Jun Soo Kwon [2,3,4]

[1] Max Planck Institute for Human Cognitive and Brain Sciences, Leipzig, Germany; [2] Department of Brain and Cognitive Sciences, College of Natural Sciences, [3] Institute of Human Behavioral Medicine, SNU-MRC, [4] Department of Psychiatry, College of Medicine, Seoul National University, Seoul, South Korea.

S1. Simulations for plausible underlying neurobiological structures

In order to suggest plausible underlying structures of our current results using a multivariate fusion analysis in the main text, we simulated 2-D data with the dimensions of 50 x 50 for 30 subjects for two groups, respectively. The 2-D images were generated by multiplying mixing weights and two sources images. The sources have two Gaussian signals (full width at half maximum (FWHM) of 10 pixels) with the maximal value of 1 at each peak. The upper peaks overlapped with a distance from 0 to 20 pixels with a bin of two pixels, and the lower peaks were constantly distant across different source configurations (the lower left peak in the source #1, the lower right peak in the source #2 in **Fig. S1A**). The mixing weights for the first source were random variables from a normal distribution of $N(0.8,0.05^2)$ for the group #1 and $N(0.5,0.05^2)$ for the group #2. The mixing weight for the second source were random variables from $N(0.35,0.07^2)$ for both groups.

After data generation with different degrees (distance between the peaks d=0, 2, 4, … , 20 pixels) of overlaps, the univariate and multivariate approaches were compared in terms of area under the curve (AUC) of receiver-operator curves (ROC) as follows:

(1) T-statistic map for group difference is computed with 30 images for each group

(2) Independent component analysis (ICA) finds two independent components (ICs) and the mixing weights are compared for group difference using two sample t-test

(3) True positive rate (TPR) and false negative rate (FNR) are computed in detecting non-zero pixels in the first source (ground truth) using the absolute image of either Z-transformed T-statistic map (univariate) or IC map with group difference in mixing coefficients (multivariate)

(4) Repeat (1)~(3) for 1000 times for each degree of overlap

For the ICA, we used FastICA algorithm [1] that is implemented in an MATLAB package (http://research.ics.aalto.fi/ica/fastica/). The summary of the simulation is given in **Fig. S1**. Detailed results of the simulations can be found in **Fig. S2**. The multivariate method performed better than the univariate method over various different configurations of the sources in terms of AUC (Fig. S1B). While the AUC of the univariate method (Fig. S1B, cyan) was constantly around (mean AUC= 0.55 ± 0.0005), the AUC of the multivariate method (Fig. S1B, magenta) increases with the increasing overlap (d=20, AUC= 0.66 ± 0.02; d=0, AUC=0.71 ± 0.01), which is due to the summation of differential and common sources at the overlapping region making it easier to detect in the IC maps.

As shown in **Fig. S1C/D**, the discrepancy between the multivariate and univariate method in terms of the magnitude of recovered sources is more pronounced especially when the differential source and the common source are overlapped to larger extent. On the other hand, the differences in spatial disposition are more exaggerated when the sources are distinct mainly due to the false positives (the regions at the upper right and lower right position) from the multivariate analysis. It suggests that the inclusion of false positives in the IC maps may be possible when the true source with differential weights between groups has no

overlaps with the common sources, which would be, however, less likely for biological signals with high spatial dependency.

Based on this simulation, we speculate that the partial overlaps between the our multivariate findings in the main text (see Results) and the univariate findings from a multi-site study [2], besides that the aims of the analyses are different, can be attributed to that the joint independent components showed significant group differences between patients with obsessive-compulsive disorder (OCD) and the healthy controls (i.e. GM #2 and FA #2 in the main text) are close to and partially overlapped with other common sources without the group differences (see **Fig. 2** in the main text). The overlaps with the common components could render the 'apparent difference' non-significant even though the significantly different contributions latently exist.

References

1. Hyvarinen A (1999) Fast and robust fixed-point algorithms for independent component analysis. Ieee Transactions on Neural Networks 10: 626-634.
2. de Wit SJ, Alonso P, Schweren L, Mataix-Cols D, Lochner C, et al. (2014) Multicenter voxel-based morphometry mega-analysis of structural brain scans in obsessive-compulsive disorder. Am J Psychiatry 171: 340-349.

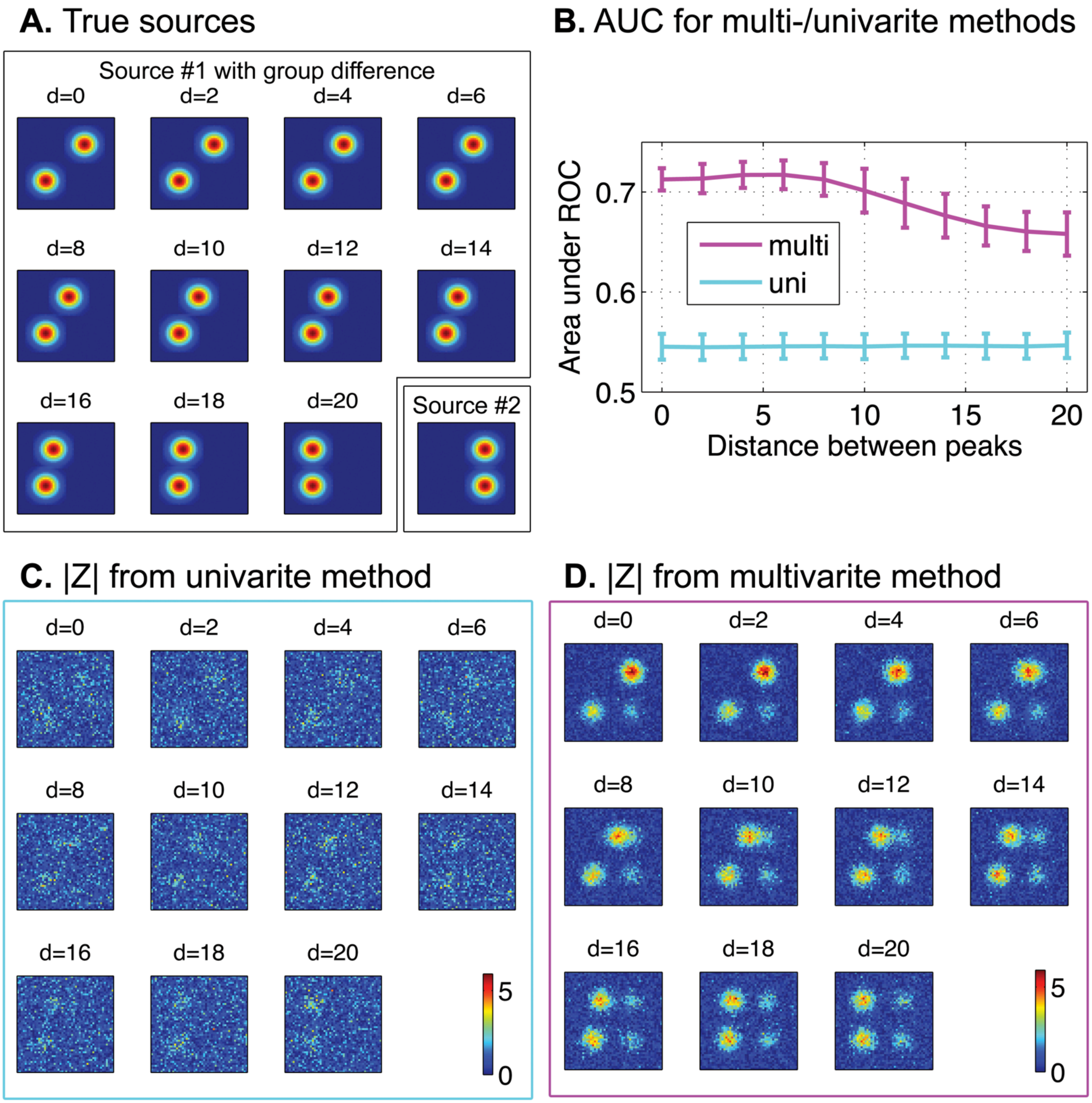

Fig. S1. Overview of simulation results. The true sources with different degree of overlaps are shown (A). Both sources have two separate Gaussian peaks and only the source #1 varies in terms of the position of the upper peak thus changing the distance between the peaks of two sources whereas the source #2 is constant. The mixing weights for two groups are generated to be different for the source #1 and similar for the source #2, thus the true group difference is always the source #1. The areas under the curves (AUCs) for the multivariate analysis (magenta) and the univariate analyses (cyan) are plotted over the distance between two peaks of different sources (B). The absolute Z-score maps from the univariate method (C) and the multivariate method (D) are given for different distances between the peaks.

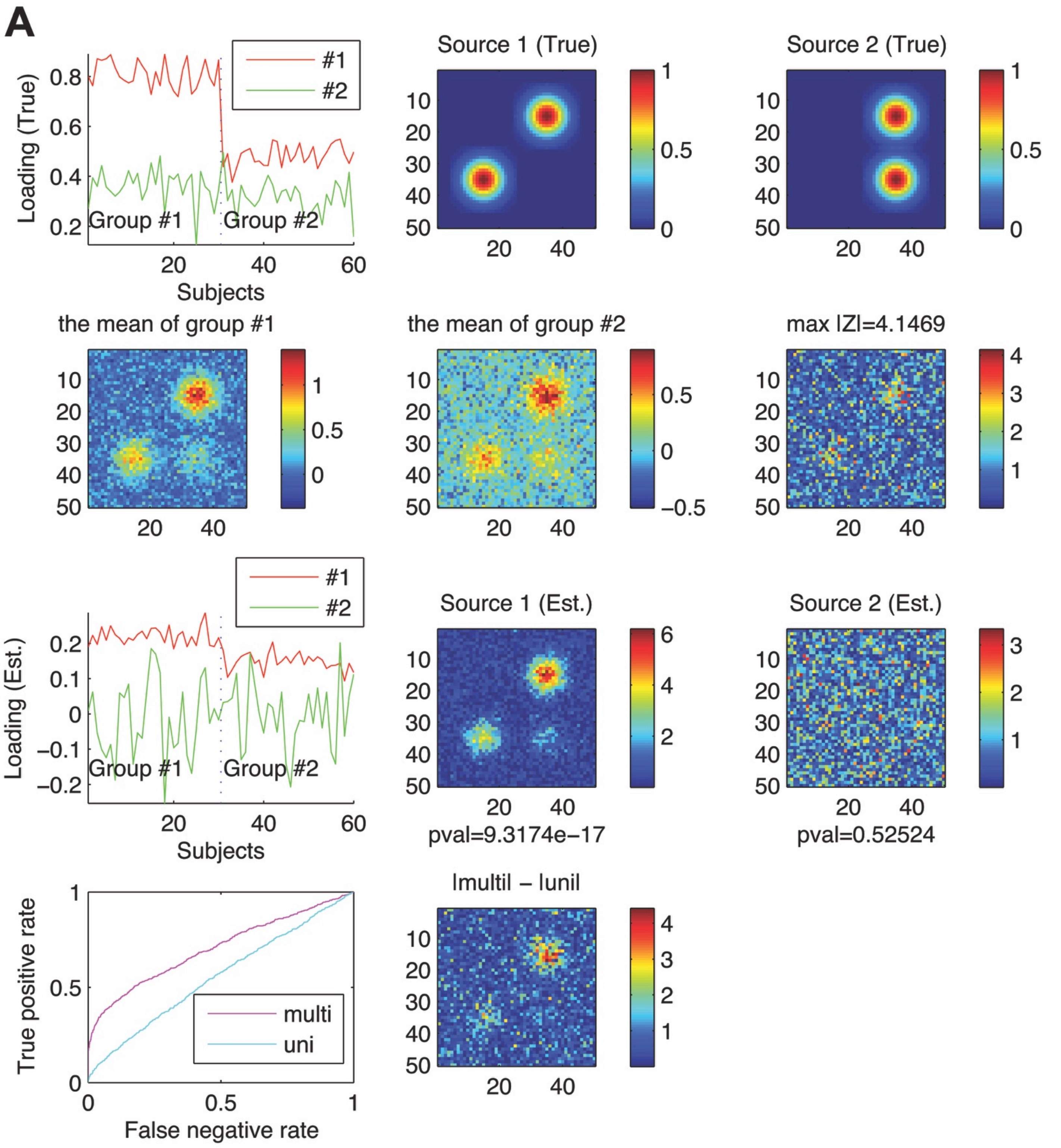

Fig. S2. An example of simulations for the sources with maximal overlaps (A, d=0 pixel),

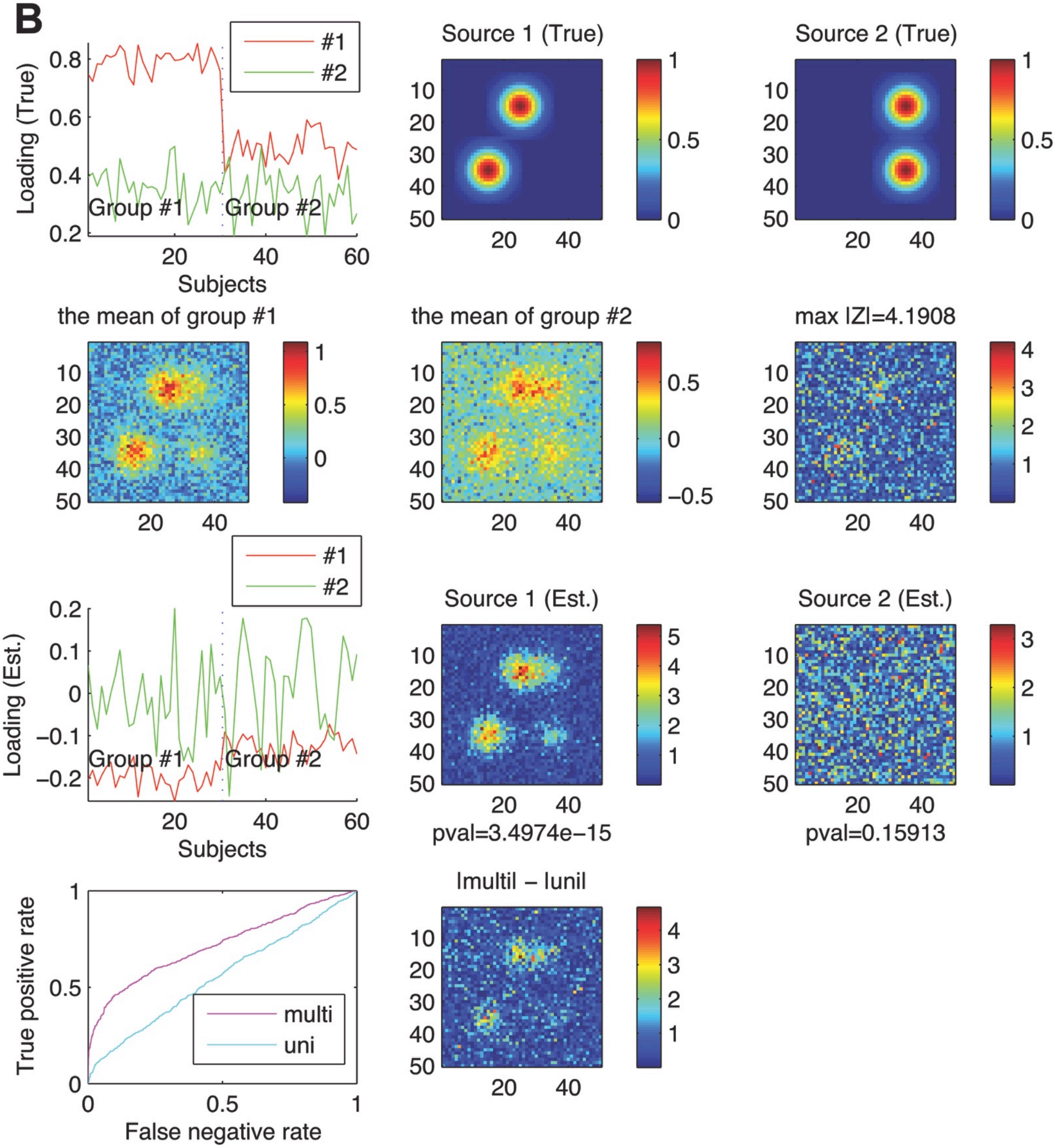

Fig. S2. (continued) an example of simulations for the sources with intermediate overlaps (B, d=10 pixel),

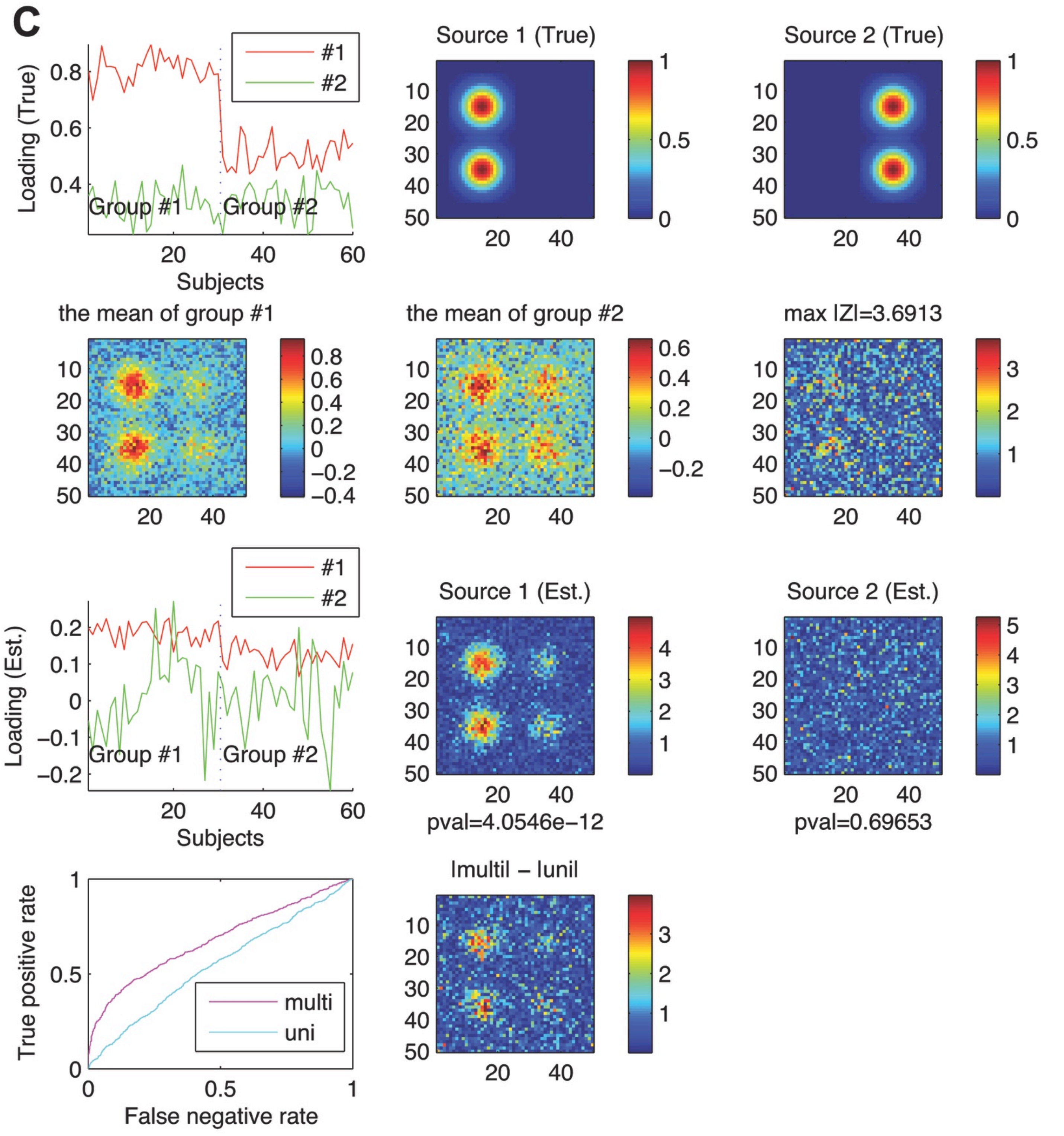

Fig. S2. (continued) and an example of simulations for the sources with minimal overlaps (C, d=20 pixels). From the top row, the true loading coefficients are plotted (first row, left) for the first group (N=30) and the second groups (N=30) for the source #1 (red) and the source #2 (green). As noted in the text, the mixing weights for the first source were random variables from a normal distribution of N (0.8, (0.05)^2) for the group #1 and N (0.5, (0.05)^2) for the group #2. The mixing weights for the second source were random variables from N (0.35, (0.07)^2) for both groups.

The true source #1 (first row, center) and source #2 (first row, right) are given in the first row as well. At the second row, the mean images are given for the group #1 (second row, left) and the group #2 (second row, center). The absolute values of the Z-transformed T-statistic maps testing for the group difference are given (second row, right). At the third row, estimated loading coefficients using the ICA method are plotted for each group (third row, left). The absolute values of the Z-transformed source maps are given for the source #1 (third row, center) and the source #2 (third row, right). Below each estimated source map, a p-value for a group difference in loading coefficients is also given. Finally at the bottom row, receiver-operator curves are given for the multivariate (magenta) and univariate (cyan) methods (fourth row, left). The absolute difference between the absolute Z-maps from multi-/univariate methods are given as well (fourth row, right).
